# Whole Saliva has a Dual Role on the Adherence of *Candida albicans* to Polymethylmetacrylate

**DOI:** 10.2174/1874210600802010001

**Published:** 2008-01-08

**Authors:** N Elguezabal, J.L. Maza, S. Dorronsoro, J. Pontón

**Affiliations:** 1 Department of Inmunología, Microbiología y Parasitología, Facultad de Medicina y Odontología, Universidad del PaísVasco, Spain; 2 Department of Estomatología, Facultad de Medicina y Odontología, Universidad del PaísVasco, Spain; 3 Department of Biología Oral, Facultad de Odontología, Universidad Nacional de Córdoba, República Argentina; 4 Department of Inmunología, Microbiología y Parasitología, Facultad de Medicina y Odontología, Universidad del PaísVasco, Spain

## Abstract

Adhesion of *Candida albicans *to acrylic of dental prostheses or to salivary macromolecules adsorbed on their surface is believed to be a critical event in the development of denture stomatitis. In previous studies our group has shown that adhesion of *C. albicans* germ tubes to polystyrene is decreased by saliva whereas *C. albicans* yeast cells adhesion to the same material is enhanced. The results presented in this study confirm this dual role played by whole saliva, since it decreased the adhesion of germ tubes but increased the adhesion of yeast cells to polymethylmetacrylate (PMMA). These effects mediated by whole saliva do not seem to be related to an inhibition of the germination of *C. albicans*, since similar levels of filamentation were observed in presence and absence of saliva. These results may give new insights into the conflicting role of saliva in the adhesion of *C. albicans* to acrylic resins of dental prostheses.

## INTRODUCTION

*Candida albicans* is a dimorphic fungus that is commensal in the gastrointestinal and reproductive tracts of healthy individuals. Under certain predisposing conditions, *C. albicans* can convert into a pathogen capable of causing a variety of oral infections including pseudomembranous candidiasis, erythematous candidiasis and hyperplasic candidiasis, as well as *Candida*-associated denture stomatitis, *Candida*-associated angular cheilitis, rhomboid glossitis and chronic mucocutaneous candidiasis [1].

*Candida*-associated denture stomatitis is an inflammatory process that affects the oral mucosa of 25-65% of patients wearing removable dental prostheses [2]. The etiology is multifactorial consisting of either ill-fitting prostheses leading to mechanical irritation or poor hygiene leading to chronic infection. Regardless of the initiating process *C. albicans* is the major cause of fungal origin in denture stomatitis [3]. The first step implicated in denture stomatitis is adherence to acrylic or to salivary pellicles adsorbed on the surface of dental prosthesis being the most important event in the ability of *C. albicans* to colonize dentures in the mouth [4].

*C. albicans* can produce biofilms on natural surfaces, such as teeth, and foreign surfaces, such as prostheses. These biofilms are normally resistant to common antimicrobial therapy an increasing problem in clinics [5].

Saliva is the biologic fluid that bathes all oral surfaces and acts as a defense against microorganisms present in the oral cavity [6]. However, contradictory results have been obtained when the effect of saliva on the adherence of *C. albicans* to acrylic and plastic has been assessed. Several studies have shown that saliva reduces the adherence of *C. albicans* to plastic [7-9], a restorative material [10,11], PMMA [12,13] and epithelial cells [14] but others have observed that saliva enhances the adherence of *C. albicans* to PMMA [15], polystyrene [4] and epithelial cells [16]. In a previous paper, we have shown that whole saliva plays a different role in the adhesion of *C. albicans* to polystyrene depending on the morphological phase of *C. albicans*, since it enhanced the adhesion to polystyrene of yeast cells but decreased the adhesion of germinated cells [9]. In the present study, we have assessed the influence of whole saliva on adherence of *C. albicans* to PMMA in an attempt to extend our previous observation to the acrylic used to make dental prostheses.

## MATERIALS AND METHODOLOGY

*C. albicans* serotype A (NCPF 3153), a filamentous strain obtained from the National Collection of Pathogenic Fungi (NCPF, Bristol, United Kingdom), *C. albicans* Ca2, a germ tube-deficient mutant of the parental strain serotype A NCPF 3153, kindly supplied by Dr. A. Cassone (Istituto Superiore di Sanita, Rome, Italy) and *C. albicans* UC1, an oral isolate from the Universidad de Cordoba (Argentina), were used in these experiments. The strains were maintained at 4 ºC on slants containing 20 g of glucose, 10 g of yeast extract, and 20 g of agar per liter.

The experimental protocols to obtain human saliva were approved by the Institutional Review Board of the School of Medicine and Odontology at the University of the Basque Country, Leioa, Spain, and the subjects gave their informed consent. Unstimulated whole saliva samples were collected and pooled from 5 healthy donors to eliminate sample variations. The donors had not taken any medication during the 3 months preceding the study and had no active periodontal disease or active caries. Saliva was centrifuged at 6000 g for 30 min at 4 ºC and the supernatant was stored at 4 ºC to be used the same day or stored at –80 ºC until used.

The acrylic strips for the adhesion assay were prepared as described by Samaranayake and MacFarlane [17], with some modifications. Briefly, transparent self-polymerizing acrylic powder (1.5 g of PMMA powder) was spread on an aluminium-foil-covered glass slide (2.5 x 7.5 cm). Monomer liquid (1ml) was poured on to the surface of the slide and immediately a second slide similar to the first was placed on top of the polymerizing mixture, and the slides were firmly secured at both ends with two binder clips. After bench curing for 30 min, the glass slides were separated. The resultant acrylic strips were cut into 5 x 5 mm squares and immersed in distilled water for 1 week to leach excess monomer. The strips were then ultrasonicated for 20 min, washed again in sterile distilled water, dried and used for the adhesion assay.

A modification of the method described by Tronchin *et al*. [18] was used in all adhesion experiments. Briefly, yeast cells were inoculated in medium 199, pH 6.7, at a final concentration of 8 x 10^5^ cells/mL and were incubated for 2 h at 37 ºC or 25 ºC in 24-well tissue culture polystyrene plates containing the PMMA pieces and 350 µL of the yeast cell suspension. After incubation, the pieces were removed from the wells, washed with saline solution, and germination was quantified by counting the total number of cells and the number of germinated cells in 12 fields (0.64 mm2 each) per PMMA square by means of a graticule mounted in the focus of the ocular. The percentage of germination was calculated by the following equation: % Germination = (germinated cells/ total cells) x 100. PMMA pieces were washed three times with saline, and the adhesion was quantified by counting the total number of cells in the same 12 fields. The percentage of adhesion was calculated by the following equation: % adhesion = (adhered cells/ total cells) x 100. All values quoted represent mean figures derived from at least 4 independent assays. To determine the effect on adherence of the salivary pellicles*,* the PMMA pieces were previously incubated in 350 µL of whole saliva for 30 min at 37 ºC.

The ANOVA test was used to assess the significance of differences between means in adherence assays. Data were considered significant at P<0.05.

## RESULTS

**Fig. (1).**
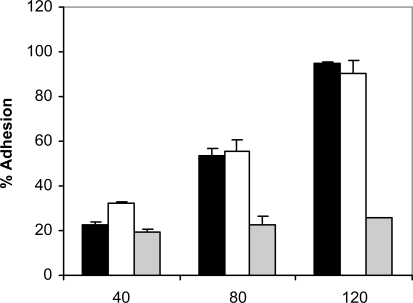
Adhesion of *C. albicans* NCPF 3153 (black), UC1 (white) and Ca2 (gray) to PMMA at 37^º^C. Results represent the means of quadruplicate determinations ±SD.

**Fig. (2).**
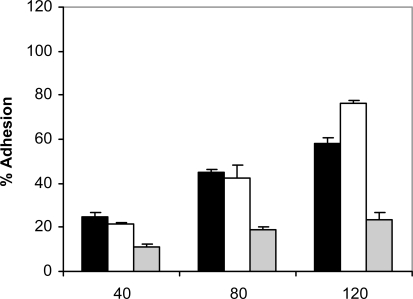
Adhesion of *C. albicans* NCPF 3153 (black), UC1 (white) and Ca2 (gray) to PMMA at 25^º^C. Results represent the means of quadruplicate determinations ±SD.

At 37 ^º^C and in the absence of human saliva, the adhesion of *C. albicans* NCPF 3153, UC1 and Ca2 to PMMA increased with time, reaching a maximum at 120 minutes (Fig. **1**). However, the levels of adhesion were not the same for the three strains since *C. albicans* Ca2 reached only a 25% of adhesion whereas *C. albicans* NCPF 3153 and UC1 exceeded 90% of adhesion. At 25 ºC (Fig. **2**), adherence of all three strains increased with time, although the percentages of adhesion reached were significantly lower than the ones observed at 37 ºC for strains *C. albicans* NCPF 3153 and UC1 (P<0.01 at 80 and 120 min) but similar to those observed for *C. albicans* Ca2.

**Fig. (3).**
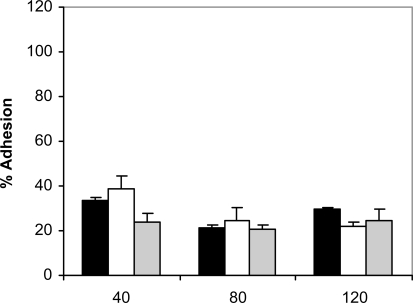
Adhesion of *C. albicans* NCPF 3153 (black), UC1 (white) and Ca2 (gray) to PMMA at 37^º^C in presence of saliva. Results represent the means of quadruplicate determinations ±SD.

Addition of saliva to the adhesion assay changed the kinetics of adhesion observed in absence of saliva (Fig. **3**). At 40 min of incubation at 37ºC, the adherence observed for all the strains was higher than the adherence in the absence of saliva at the same period of time, with a rise in adhesion ranging from 11.4% (*C. albicans* NCPF 3153) to 4.2% (*C. albicans* Ca2). However, at 80 and 120 min in the presence of saliva the adhesion of *C. albicans* to PMMA was significantly reduced (P<0.001).

**Fig. (4).**
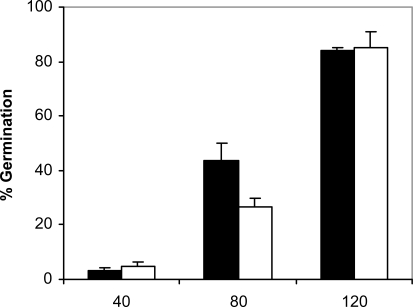
Germination of *C. albicans* NCPF 3153 (black) and UC1 (white) to PMMA at 37^º^C. Results represent the means of quadruplicate determinations ±SD.

**Fig. (5).**
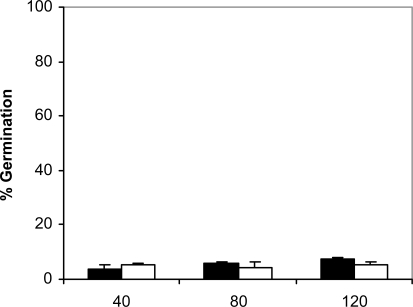
Germination of *C. albicans* NCPF 3153 (black) and UC1 (white) to PMMA at 25^º^C. Results represent the means of quadruplicate determinations ±SD.

**Fig. (6):**
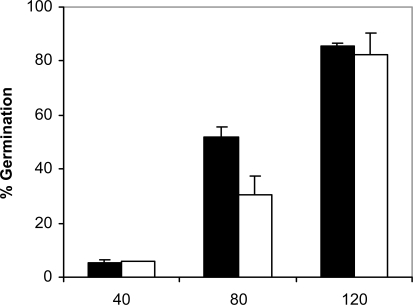
Germination of *C. albicans* NCPF 3153 (black) and UC1 (white) to PMMA at 37^º^C in presence of saliva. Results represent the means of quadruplicate determinations ±SD.

Since adhesion of *C. albicans* to plastic materials has been shown to be related with germination [18,19], we assessed the filamentation in the conditions of the adhesion assay. At 37ºC and in absence of saliva, germination increased with time in *C. albicans* NCPF 3153 and UC1 (Fig. **4**). At 40 min of incubation the majority of the *C. albicans* cells presented yeast morphology and only 3% showed short germ tubes. Longer periods of incubation produced an increase in the percentage of germination and an extension in the length of the germ tubes. The maximum level of germination, which was similar for both strains reaching 85% at 120 min. However, germination at 25ºC (Fig. **5**) was very low and remained fairly constant through the time of incubation studied (4-7%). As expected, *C. albicans* Ca2 did not germinate at 25 or 37ºC in presence or absence of saliva (data not shown). In the presence of saliva (Fig. **6**), germination also increased with time showing levels similar to those reached by the controls in absence of saliva.

## DISCUSSION

*C. albicans* adheres to a variety of surfaces in the oral cavity that are constantly bathed by saliva [6]. Saliva has a defensive role in the oral cavity that is non-specific (mucins, lysozyme, peroxydase, histatins, etc.) and specific (secretory IgA). Mucins enhance agglutination preventing colonization [20, 21] whereas lysozyme, peroxydase [6] and histatins [22, 23] are candidacidal. On the other hand, saliva provides water, nutrients and adherence factors [6]. A number of studies have shown that the adherence of *C. albicans* to acrylic materials [4,7-9,15] can be modulated by saliva. However, the precise role of saliva on the adhesion of *C. albicans* to acrylic is not clear, since both an increase and a decrease in adhesion have been described [4,7,8]. A possible explanation for this contradictory effect has been proposed by San Millán *et al*. [9] when studying the adhesion of *C. albicans* to polystyrene, since whole saliva decreased or enhanced the adhesion of *C. albicans* to polystyrene depending on the morphological phase of *C. albicans*. Thus, it is possible that whole saliva plays a dual role on the adhesion of *C. albicans* to plastic materials used to make dental prostheses, decreasing adhesion of germinated cells and enhancing the adhesion of yeast cells.

The results presented in this study confirm this dual role played by whole saliva, since it decreased the adhesion of germ tubes to PMMA but increased the adhesion of yeast cells to the acrylic. Although germination is an important factor in the adhesion of *C. albicans* to plastic surfaces and epithelial cells [18, 24] and in this study we have observed that adhesion increases in parallel with germ tube formation, the effects mediated by whole saliva do not seem to be related to an inhibition of the germination of *C. albicans*, since similar levels of filamentation were observed in presence and absence of saliva. Secretory IgA and possibly other salivary components are likely to modulate the adhesion of *C. albicans* to PMMA by binding the cell wall surface of the fungus. In the inhibitory effect, binding of the cell wall will mask the fungal adhesins, while the enhancement of adhesion may be mediated by a reorganization of the cell wall adhesins induced by certain types of antibodies [10,11,19]. Interestingly, the dual effect caused by whole saliva may be specific of plastic materials since it was not observed with the composite Herculite, where only the inhibitory effect on the adhesion of *C. albicans*was observed [11].

Future studies should be aimed at finding antimicrobial agents that can decrease *C. albicans* adhesion to PMMA or kill *C. albicans* adhered to PMMA surface. In this sense, more studies like the one performed recently by Manfredi *et al*. [25] showing that a synthetic killer peptide has potential candidacidal effect on *C. albicans* cells adhered to acrylic surfaces should be done.

## CONCLUSIONS

In conclusion, the results presented in this study demonstrate that whole saliva decreases the adherence of *C. albicans* to PMMA, although our results show it plays a dual role that depends on the morphological phase of the fungus.

## ABBREVIATIONS

PMMA: = Polymethylmetacrylate
NCPF: = National Collection of Pathogenic Fungi
SD: = Standard deviation

## References

[1] Aguirre JM (2002). Candidiasis orales. Rev Iberoam Micol.

[2] Budtz-Jörgensen E (1990). Etiology, pathogenesis, therapy and prophylaxis of oral yeast infections. Acta Odontol Scand.

[3] Budtz-Jörgensen E (1974). The significance of *Candida albicans* in denture stomatitis. Scand J Dent Res.

[4] Vasilas A, Molina L, Hoffman M, Haidaris C (1992). The influence of morphological variation on *Candida albicans* adhesion to denture acrylic in vitro. Arch Oral Biol.

[5] Douglas LJ (2003). Candida biofilms and their role in infection. Trends Microbiol.

[6] Marcotte H, Lavoie MC (1998). Oral microbial ecology and the role of salivary immunoglobulin A. Microbiol Mol Bio Rev.

[7] McCourtie J, MacFarlane T, Samaranayake L (1986). Effect of saliva and serum on the adherence of Candida spp to clorhexidine-treated denture acrylic. J Med Microbiol.

[8] Samaranayake LP, McCourtie J, MacFarlane TW (1980). Factors affecting the in vitro adherence of *Candida albicans* to acrylic surfaces. Arch Oral Biol.

[9] San Millán R, Elguezabal N, Regúlez P, Moragues MD, Quindós G, Pontón J (2000). Effect of salivary secretory IgA on the adhesion of *Candida albicans* to polysterene. Microbiology.

[10] Elguezabal N, Maza JL, Pontón J (2004). Inhibition of adherence of *Candida albicans* and *Candida dubliniensis* to a resin composite restorative dental material by salivary secretory IgA and monoclonal antibodies. Oral Dis.

[11] Maza JL, Elguezabal N, Prado C, Ellacuría J, Soler I, Pontón J (2002). *Candida albicans* adherence to resin-composite restorative dental material: Influence of whole human saliva. Oral Surg Oral Med Oral Pathol.

[12] Silva Moura J, da Silva WJ, Pereira T, Del Bel Curry AA, Rodrigues-Garcia RC (2006). Influence of acrylic resin polymerization methods and saliva on the adherence of four Candida species. J Prosthet Dent.

[13] Karaagaclioglu L, Gulsen C, Yilmaz B, Ayham N, Semiz O, Levent H (Aug1). The adherence of *Candida albicans* to acrylic resin reinforced with different fibers. J Mater Sci Mater Med Epub ahead of print.

[14] Umazume M, Ueta E, Osaki T (1995). Reduced inhibition of *Candida albicans* adhesion by saliva from patients receiving oral cancer therapy. J Clin Microbiol.

[15] Edgerton M, Scannapieco F, Reddy M, Levine M (1993). Human submandibular-sublingual saliva promotes adhesion of *Candida albicans* to polymethylmethacrylate. Infect Immun.

[16] Holmes AR, Bandara BMK, Cannon RD (2002). Saliva promotes *Candida albicans* adherence to human epithelial cells. J Dent Res.

[17] Samaranayake LP, MacFarlane TW (1980). An in vitro study of the adherence of *Candida albicans* to acrylic surfaces. Arch Oral Biol.

[18] Tronchin G, Bouchara JP, Robert R, Senet JM (1988). Adherence of *Candida albicans* germ tube to plastic: ultrastructural and molecular studies of fibrillar adhesins. Infect Immun.

[19] San Millán R, Ezkurra PA, Quindós G, Robert R, Senet JM, Pontón J (1996). Effect of monoclonal antibodies directed against *Candida albicans* cell wall antigens on the adhesion of the fungus to polystyrene. Microbiology.

[20] Hoffman MP, Haidaris CG (1993). Analysis of *Candida albicans* adhesion to salivary mucin. Infect Immun.

[21] Tabak LA, Levine MJ, Mandel ID, Ellison SA (1982). Role of salivary mucin in protection of the oral cavity. J Oral Pathol.

[22] Fitzgerald DH, Coleman DC, O´Connell BC (2003). Susceptibility of *Candida dubliniensis* to salivary histatin 3. Antimicrob Agents Chemother.

[23] Oppenheim FG, Xu T, McMillan FM, Levitz SM, Diamond RD, Offner GD, Troxler RF (1988). Histatins, a novel family of histidine-rich proteins in human parotid secretions. J Biol Chem.

[24] Kimura LH, Pearsall NN (1980). Relationship between germination of *Candida albicans* and increased adherence to human buccal epithelial cells. Infect Immun.

[25] Manfredi M, Merigo E, Salati A, Conti S, Savi A, Polonelli L, Bonanini M, Vescovi P (2007). In vitro candidacidal activity of a synthetic killer decapeptide (KP) against *Candida albicans* cells adhered to resin acrylic discs. J Oral Pathol Med.

